# Novel Bivalent mRNA‐LNP Vaccine for Highly Effective Protection against Pneumonic Plague

**DOI:** 10.1002/advs.202501286

**Published:** 2025-04-25

**Authors:** Uri Elia, Yinon Levy, Hila Cohen, Ayelet Zauberman, David Gur, Inbal Hazan‐Halevy, Moshe Aftalion, Shani Benarroch, Erez Bar‐Haim, Orit Redy‐Keisar, Ofer Cohen, Dan Peer, Emanuelle Mamroud

**Affiliations:** ^1^ Department of Biochemistry and Molecular Genetics Israel Institute for Biological Research Ness‐Ziona 76100 Israel; ^2^ Laboratory of Precision NanoMedicine Shmunis School for Biomedicine and Cancer Research George S. Wise Faculty of Life Sciences Tel Aviv University Tel Aviv 69978 Israel; ^3^ Center for Nanoscience and Nanotechnology Tel Aviv University Tel Aviv 69978 Israel; ^4^ Department of Materials Sciences and Engineering Iby and Aladar Fleischman Faculty of Engineering Tel Aviv University Tel Aviv 69978 Israel; ^5^ Cancer Biology Research Center Tel Aviv University Tel Aviv 69978 Israel; ^6^ Department of Organic Chemistry Israel Institute for Biological Research Ness‐Ziona 76100 Israel

**Keywords:** lipid nanoparticles, mRNA, vaccine, *Y. pestis*

## Abstract

*Yersinia pestis*, the causative agent of plague, remains a significant global health hazard and a potential top‐tier biothreat despite modern medical advances. Here, two mRNA constructs encoding different versions of the low‐calcium response virulence (LcrV) protective antigen, an essential virulence factor of *Y. pestis*, are designed and evaluated. Next, the immunogenicity and protective efficacy both independently and in combination is assessed with the previously reported F1‐encoding mRNA construct in the well‐established mouse model of pneumonic plague. The findings reveal that human Fc‐conjugated F1 + LcrV combination mRNA vaccination resulted in significant immune activation and substantial protection against intranasal *Y. pestis* challenge. Notably, the combined vaccine demonstrates protective efficacy against two highly virulent wild‐type *Y. pestis* strains representing distinct biovars and an atypical, unencapsulated strain. This study represents the first comprehensive evaluation of mRNA constructs encoding innovatively designed versions of LcrV and F1 for pneumonic plague prevention, addressing critical gaps in current vaccination approaches. This study establishes the mRNA‐lipid nanoparticle (LNP) platform as a promising tool for addressing bacterial pathogens, including those resistant to antibiotics. By broadening its applicability to diverse threats, this technology represents an innovative approach to tackling some of the most pressing challenges in global health.

## Graphical abstract

The study investigates mRNA‐lipid nanoparticle vaccines targeting *Yersinia pestis*, the causative agent of plague. Combined F1+LcrV mRNA vaccination elicited robust immune responses and protected mice against virulent wild‐type and atypical strains. This analysis underscores the potential of the mRNA‐lipid nanoparticle platform to combat bacterial pathogens, address antibiotic resistance, and contribute to innovative global health solutions.

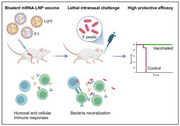

## 1 Introduction

Plague, caused by the Gram‐negative bacterium *Yersinia pestis*, has been one of the most devastating diseases throughout human history, responsible for three major pandemics including the catastrophic Black Death in the Middle Ages.^[^ ^1^ ^]^ Although modern medicine has largely contained plague through antibiotics and stringent public health measures, the threat of antibiotic‐resistant strains makes it even more important to develop alternative preventive and therapeutic approaches. Recent outbreaks, such as the one in Madagascar in 2017, underscore that plague is not just a disease of the past but continues to pose a significant threat today. The high mortality rates and potential for person‐to‐person transmission classify *Y. pestis* as a top‐tier biothreat, emphasizing the need for effective and quickly producible vaccines to prevent both natural and deliberate outbreaks of pneumonic plague, which can lead to rapid respiratory failure and death if not treated promptly.^[^ ^2^, ^3^, ^4^, ^5^ ^]^

As of now, there are no licensed vaccines available for *Y. pestis* in Western countries.^[^ ^6^ ^]^ Current investigational subunit vaccines targeting plague have predominantly utilized the F1 capsule antigen and the low‐calcium response virulence (LcrV or V) protein, both of which play important roles in enhancing the bacterium's virulence and facilitating its evasion of host immune responses. The F1 antigen forms the bacterial capsule, shielding it from phagocytosis by host immune cells, while the LcrV protein acts as part of the Type III secretion system, suppressing the host's inflammatory response.^[^ ^5^ ^]^

We have recently shown that mRNA vaccines, traditionally used for viral antigens and cancer, can be effectively adapted to encode bacterial antigens like F1, offering a versatile and powerful tool for combating bacterial infectious diseases with high adaptability and response speed. Specifically, we have shown that an mRNA‐lipid nanoparticle (LNP) vaccine encoding the F1 antigen effectively protects against the bubonic form of plague in animal models.^[^ ^7^ ^]^

This study pioneers the application of the mRNA‐LNP vaccine platform to combat pneumonic bacterial diseases, with a specific focus on pneumonic plague. For the first time, we conducted a comprehensive evaluation of multiple mRNA constructs encoding distinct, innovatively designed versions of LcrV, optimizing their immunogenicity and protective efficacy. These constructs were tested both as standalone components and in combination with F1‐encoding mRNA in three well‐established mouse models. Through rigorous in vivo studies, we observed not only significant immune activation but also substantial protection against lethal *Y. pestis* challenge, emphasizing the robustness of our approach. Remarkably, our vaccine strategy demonstrated protective efficacy against two highly virulent wild‐type strains of *Y. pestis* representing distinct biovars of plague: Orientalis and Medievalis, as well as against an atypical, unencapsulated strain. These findings underscore the broad‐spectrum nature of the immune response elicited by our platform and lay the foundation for future translational studies and potential applications against other bacterial pathogens.

## 2 Results

### 2.1 Design of a LcrV‐Coding mRNA‐LNPs

The LcrV protein is considered a major, protective antigen against *Y. pestis* infection.^[^ ^8^, ^9^ ^]^ We sought to investigate the feasibility of expressing the V antigen using the mRNA‐LNPs platform and to examine its protective potential in the mouse model of pneumonic plague. In our previous study,^[^ ^7^ ^]^ conducted on the capsule F1 antigen, we showed that structural modifications of the original bacterial F1 antigen are sufficient for proper protein expression in the mammalian background that promoted a protective response. Furthermore, we also demonstrated that conjugation of the encoded monomeric F1 protein to the human Fc (hFc) immunoglobulin portion, resulted in enhancement of the immune response raised against the expressed protein and ultimately provided improved protection against a lethal bubonic plague challenge.

**Figure 1:**
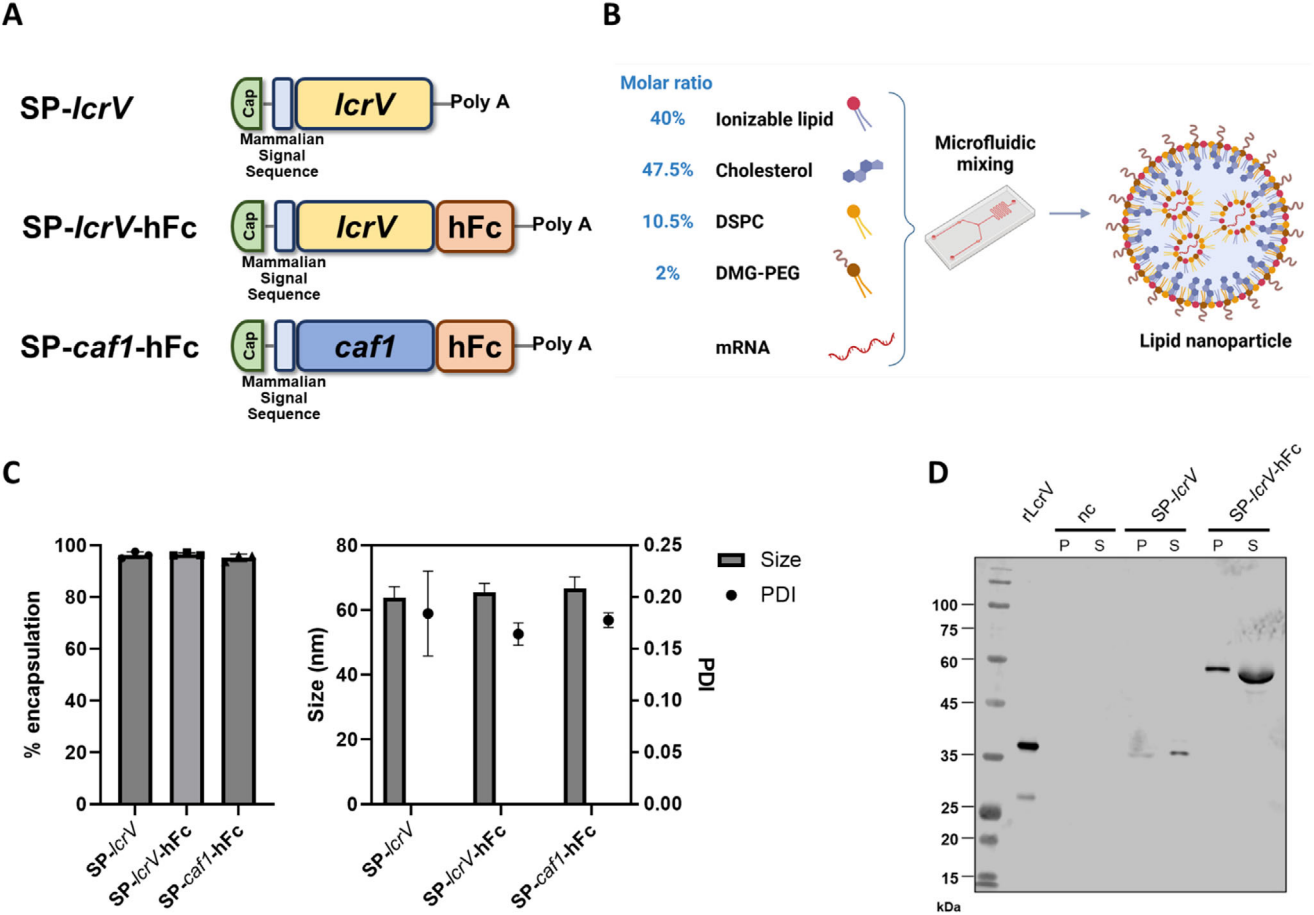
Design and synthesis of mRNA‐LNPs used in the study, physicochemical characteristics and in vitro expression. A) Schematic representation of mRNA constructs utilized in the study. Elements include: Cap (Cap), signal peptide (SP), *lcrV* gene sequence (*lcrV*), *caf1* gene sequence (*caf1*), human Fc (hFc), poly adenylation (poly A). B) Schematic of mRNA‐LNP formulation (see materials and methods section for more detail). C) Physicochemical characterization of mRNA‐LNPs used in this study, showing average RNA encapsulation efficiency (left panel) and particle size and poly dispersity index (PDI, right panel). D) Western blot analysis of LcrV expression in HeLa cells transfected with the indicated mRNA constructs for 48 h. Recombinant LcrV served as size control. Protein expression was evaluated in both cell pellet (P) and supernatant (S). nc = negative control (untransfected cells). The schematic for panel 1B was created with Biorender.

Recapitulating this strategy, we designed two mRNA constructs: In the first construct, a mammalian signal peptide originating from the human Ig kappa light chain was introduced upstream to the *lcrV* gene, resulting in the SP‐*lcrV* mRNA construct. The second construct, SP‐*lcrV*‐hFc, was designed to express a hFc‐conjugated LcrV protein, and was based on previous reports demonstrating that conjugation of proteins to hFc resulted in increased immunogenicity, half‐life and stability^[^ ^10^, ^11^, ^12^ ^]^ (see construct schematic in **Figure** **1**A). Each mRNA construct was encapsulated in an LNP formulation previously fabricated in our lab (Figure 1B), demonstrating efficient delivery of the encoding mRNA.^[^ ^13^ ^]^ Physicochemical characterization of the mRNA‐LNPs revealed a ≈60–70 nm size range, with a poly dispersity index (PDI) of <0.2, and >95% encapsulation efficiency (Figure 1C). In vitro protein expression was confirmed by transfecting HeLa cells with the different mRNA constructs, followed by Western blot analysis of supernatant and cell pellet fractions for evaluation of LcrV expression (Figure 1D).

### 2.2 Vaccination with LcrV mRNA‐LNPs Protects Both Inbred and Outbred Mouse Strains Against a Lethal Intranasal *Y. pestis* Challenge

**Figure 2:**
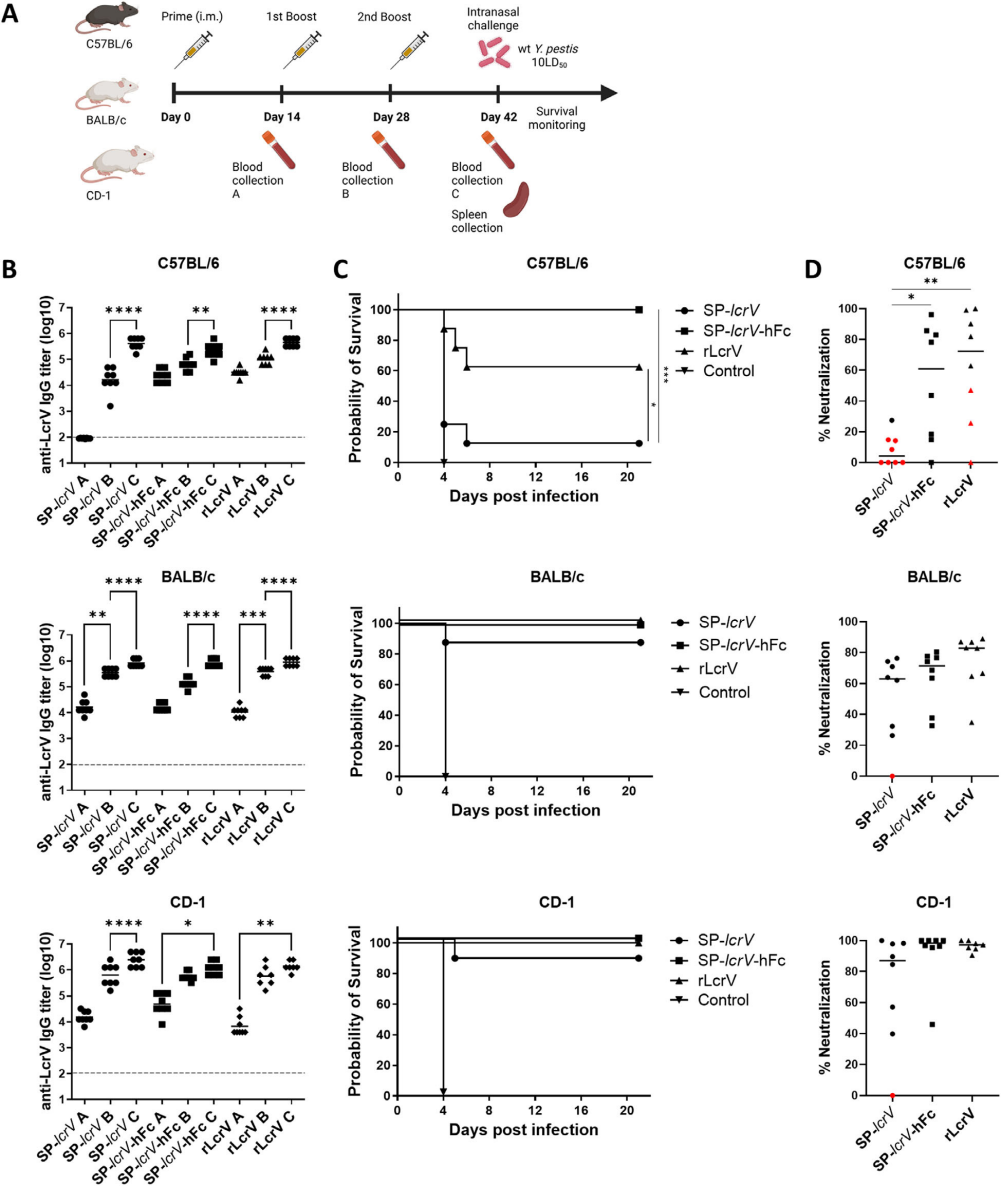
Immunogenicity and protective efficacy of LcrV against pneumonic *Y. pestis* in different mouse strains. A) Schematic representation of vaccination regimen. C57BL/6, BALB/c and CD‐1 mice (*n* = 8 / strain) were vaccinated intramuscularly with either SP‐*lcrV* (5µg), SP‐*lcrV*‐hFc (5µg) or rLcrV (80 µg) at days 0, 14 and 28. Blood samples were collected before each booster dose (denoted as A, B, abd C), and serum samples were assayed for anti‐LcrV antibodies by ELISA B). Two weeks after the last vaccination (day 42), animals were subjected to a lethal (10LD_50_) intranasal Kimberley53 challenge and monitored for survival C). D) Sera from vaccinated mice were tested for cytotoxicity neutralization (% neutralization). Surviving animals are represented by black symbols and non‐surviving animals are represented by red symbols. Mean % neutralization is indicated by horizontal black lines. Statistical analysis was performed using a one‐way ANOVA with Tukey's multiple comparisons test (for immune responses and neutralization assay) or log‐rank (Mantel‐Cox) test (for survival plot), (*p < 0.05, **p < 0.01, ***p < 0.001, and ****p < 0.0001). Dashed line indicates limit of detection for ELISA. The schematic for panel 2A was created with Biorender.

To evaluate the immunogenicity of LcrV mRNA‐LNPs, inbred C57BL/6 mice were vaccinated with either SP‐*lcrV* or SP‐*lcrV*‐hFc thrice at two‐weeks intervals, and anti‐V IgG antibody titers were measured 14 days after each administration (see outline in **Figure** **2**A). Although ultimately reaching a similar level after the third administration, anti‐LcrV titers development in the two groups displayed different kinetics (Figure 2B). While SP‐*lcrV* vaccination resulted in substantial anti‐LcrV titers only after the 2^nd^ administration, SP‐*lcrV*‐hFc exhibited faster kinetics, with high anti‐LcrV antibody titers already after the prime administration. mRNA‐elicited antibody response following the third administration was comparable to that of the recombinant protein, rLcrV. The protective efficacy of the mRNA‐LNPs against pneumonic plague was then evaluated by a challenge study, in which a lethal dose (10LD_50_; 11 000 CFU) of virulent Kimberley53 strain was administered intranasally. Figure 2C shows that C57BL/6 mice vaccinated with SP‐*lcrV*‐hFc were fully protected against the lethal challenge. In contrast, vaccination with SP‐*lcrV* afforded very little protection, with only 12.5% (1 animal) survival, despite the high anti‐LcrV IgG titers measured prior to the challenge (Figure 2B). Notably, C57BL/6 mice vaccinated with the recombinant protein were only partially protected, with 60% of the animals surviving the challenge. In order to determine whether these observations are strain‐related, we performed a similar vaccination study with inbred BALB/c and outbred CD‐1 mice (Figure 2A). As can be seen in Figure 2B, the development of anti‐LcrV IgG antibodies in both BALB/c and CD‐1 mice differed from that observed in C57BL/6 mice, as high titers in both SP‐*lcrV* and SP‐*lcrV*‐hFc groups were measured already after the prime vaccination. A significant boosting effect following the subsequent administrations was also observed. Once again, the antibody response elicited by the mRNA was comparable to that induced by the recombinant LcrV protein. Interestingly, and in contrast to the differential response observed in C57BL/6 mice, both SP‐*lcrV* and SP‐*lcrV*‐hFc vaccinated BALB/c and CD‐1 mice were highly protected against the lethal Kimberley53 challenge, with survival rates of 85.7% (BALB/c SP‐*lcrV*), 100% (BALB/c SP‐*lcrV*‐hFc), 85.7% (CD‐1 SP‐*lcrV*) and 100% (CD‐1 SP‐*lcrV*‐hFc) (Figure 2C). Additionally, vaccination of BALB/c and CD‐1 mice with the recombinant LcrV antigen led to full protection, in contrast to the partial protection afforded by rLcrV in C57BL/6 mice. We have previously developed a highly sensitive in vitro macrophage cytotoxicity neutralization assay that enables the calculation of the neutralizing capacity of anti‐LcrV serum antibodies as an in vitro correlate of plague protective immunity in mice.^[^ ^14^ ^]^ Figure 2D depicts the neutralizing capacity of serum from C57BL/6, BALB/c and CD‐1 mice vaccinated with three doses of SP‐*lcrV* mRNA, SP‐*lcrV*‐hFc mRNA, or rLcrV, and indicates individual challenge outcome (live – black /dead – red). All surviving BALB/c and CD‐1 mice displayed a strong neutralizing capacity, with the majority of animals exhibiting over 60% neutralization in the macrophage cytotoxicity assay, regardless of the vaccination type. The only BALB/c and CD‐1 mice that succumbed to the challenge were vaccinated with SP‐*lcrV* mRNA and showed no neutralizing antibodies (Figure 2D). A different trend, however, was detected in C57BL/6 mice. Although total binding antibody levels were high in all vaccination groups (Figure 2B), their general neutralizing capacity was lower compared to BALB/c and CD‐1 mice. This effect was most pronounced in C57BL/6 mice vaccinated with SP‐*lcrV*, where 7/8 animals exhibited marginal neutralizing capacity (<20%) and accordingly succumbed to the lethal challenge. Vaccination of C57BL/6 mice with recombinant LcrV was more effective at eliciting neutralizing antibodies, as only 3 out of 8 animals succumbed to the infection, displaying lower (<50%) neutralizing capacity. Interestingly, vaccination of C57BL/6 mice with SP*‐lcrV*‐hFc mRNA resulted in a wide range of neutralizing capacity, with half of the animals exhibiting <20% and the other half >75% neutralization. Remarkably, all animals in this group survived the lethal infection, highlighting the enhanced immunogenicity provided by conjugation of the LcrV to hFc. The survival of animals displaying low neutralizing capacity (<20%) of anti‐LcrV antibodies suggest that the mRNA‐LNP vaccine platform may have activated additional protective mechanisms such as cell‐mediated immunity.

### 2.3 LcrV mRNA‐LNPs Vaccination Elicits Cell‐Mediated Immune Responses

**Figure 3:**
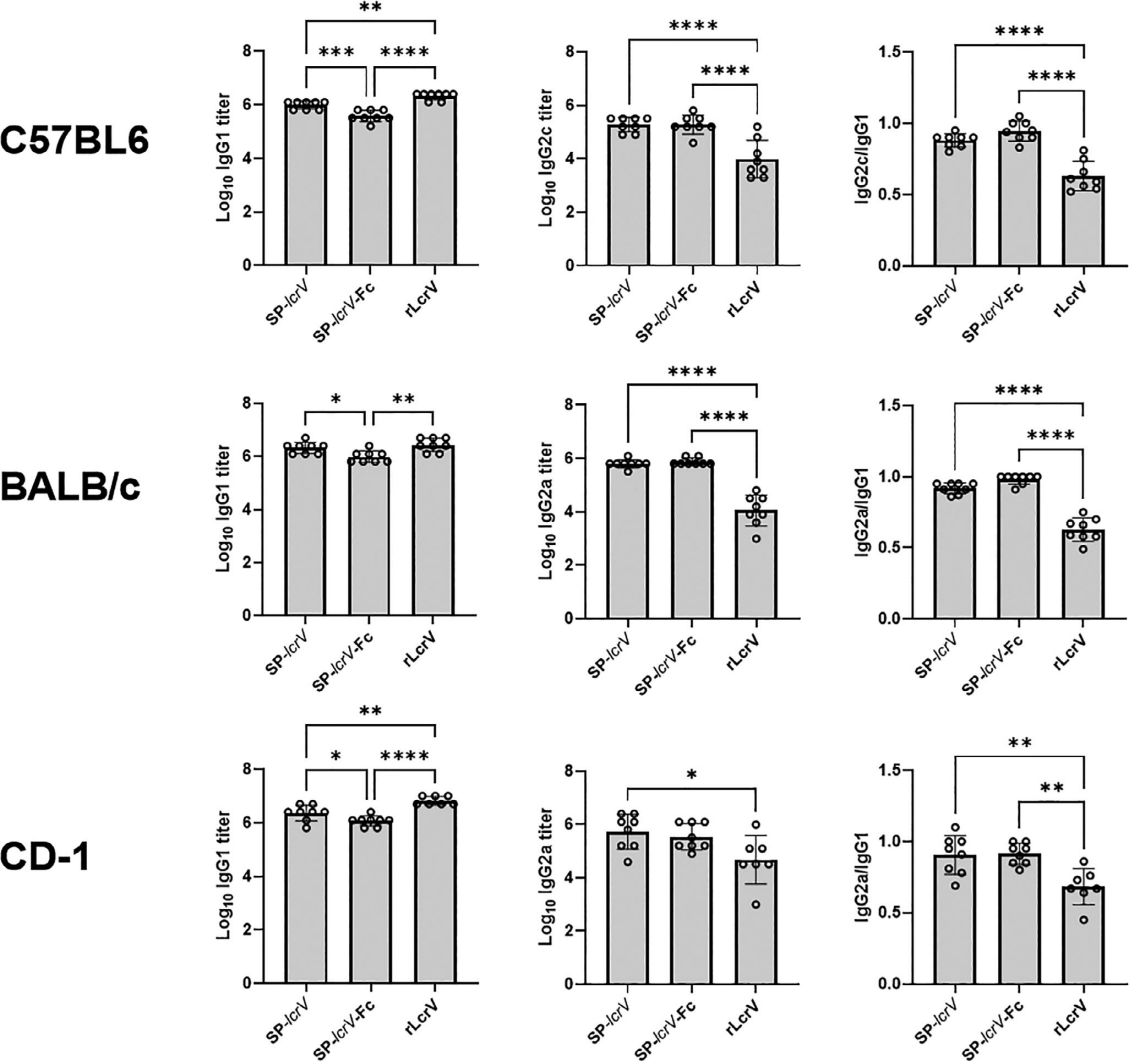
Induction of anti‐LcrV IgG subclasses by mRNA or recombinant protein vaccine. Sera from BALB/c, CD‐1 and C57BL/6 mice immunized thrice (*n* = 8) with either SP‐*lcrV* (5µg), SP‐*lcrV*‐hFc (5µg) or rLcrV (80 µg) were set to detect subclasses of anti‐LcrV specific IgGs. Statistical analysis was performed using a one‐way ANOVA with Tukey's multiple comparisons test (*p < 0.05, **p < 0.01, ***p < 0.001, and ****p < 0.0001).

To further characterize the immune responses elicited by the different vaccination groups, we evaluated the induction of specific IgG subclasses following immunization. ELISA analysis of IgG subclasses IgG1 and IgG2a (BALB/c and CD‐1) or IgG2c (C57BL/6) revealed a similar trend in all three mouse strains, with both mRNA vaccines SP‐*lcrV* and SP‐*lcrV*‐hFc resulting in a balanced IgG2a/IgG1 or IgG2c/IgG1 ratios of ≈1. In contrast, when mice of all three strains were vaccinated with the recombinant protein, they exhibited a significantly lower IgG2/IgG1 ratio (**Figure** **3**). Overall, these results suggest that vaccination with the Alum‐absorbed recombinant LcrV protein resulted in a T_H_2‐skewed response, characterized predominantly by a strong humoral response and supported by the lower IgG2/IgG1 ratio, whereas the mRNA‐based vaccination resulted in a more pronounced T_H_1 response, as evidenced by the more equitable IgG2/IgG1 ratio, suggesting the involvement of cellular immunity.

**Figure 4:**
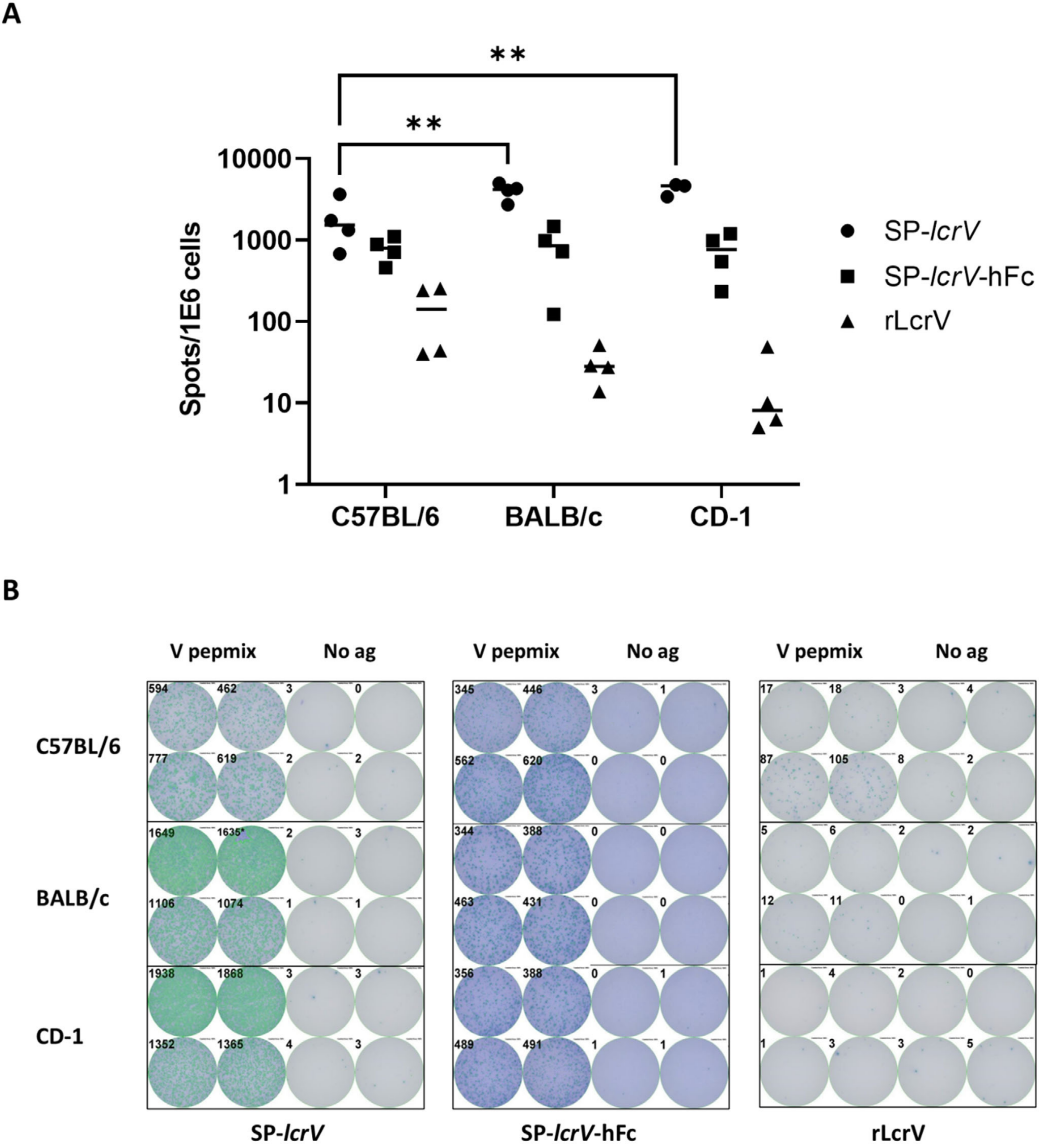
Induction of antigen‐specific cellular immunity by LcrV mRNA vaccine. Spleens were collected from SP‐*lcrV*, SP‐*lcrV‐*hFc or rLcrV vaccinated animals (*n* = 3‐4) 14 days after the last vaccine administration. Antigen‐specific cellular response was determined by ELISpot assay by quantification of IFN‐γ secreting cells A). Panel B shows representative wells from the ELISpot assay, used for quantification. Statistical analysis was performed using a two‐way ANOVA with Bonferroni's multiple comparisons test (*p < 0.05, **p < 0.01).

To directly examine the involvement of cellular immune response in the different vaccination groups, splenocytes from SP*‐lcrV* mRNA, SP*‐lcrV*‐hFc mRNA or rLcrV vaccinated BALB/c, C57BL/6 and CD‐1 mice (see vaccination outline in Figure 2A) were stimulated with an overlapping 15‐mer peptide mix covering the entire LcrV protein, and IFN‐γ secretion by individual splenocytes was recorded using ELISpot assay. As can be seen in **Figure** **4**A,B, vaccination with SP*‐lcrV* mRNA and SP*‐lcrV*‐hFc mRNA resulted in a robust IFN‐γ secretion in all three mouse strains, with a significantly stronger response in SP*‐lcrV*‐vaccinated BALB/c and CD‐1 mice compared to C57BL/6 mice. IFN‐γ secretion in SP*‐lcrV*‐hFc mRNA‐vaccinated animals was similar in all three tested mouse strains. In accordance with previous reports, vaccination with AlOH‐adsorbed rLcrV protein failed to induce substantial IFN‐γ release in all three mouse strains while inducing a strong humoral response.^[^ ^15^ ^]^

### 2.4 A Bivalent F1 + LcrV mRNA Vaccine Protects Against Pneumonic Plague

**Figure 5:**
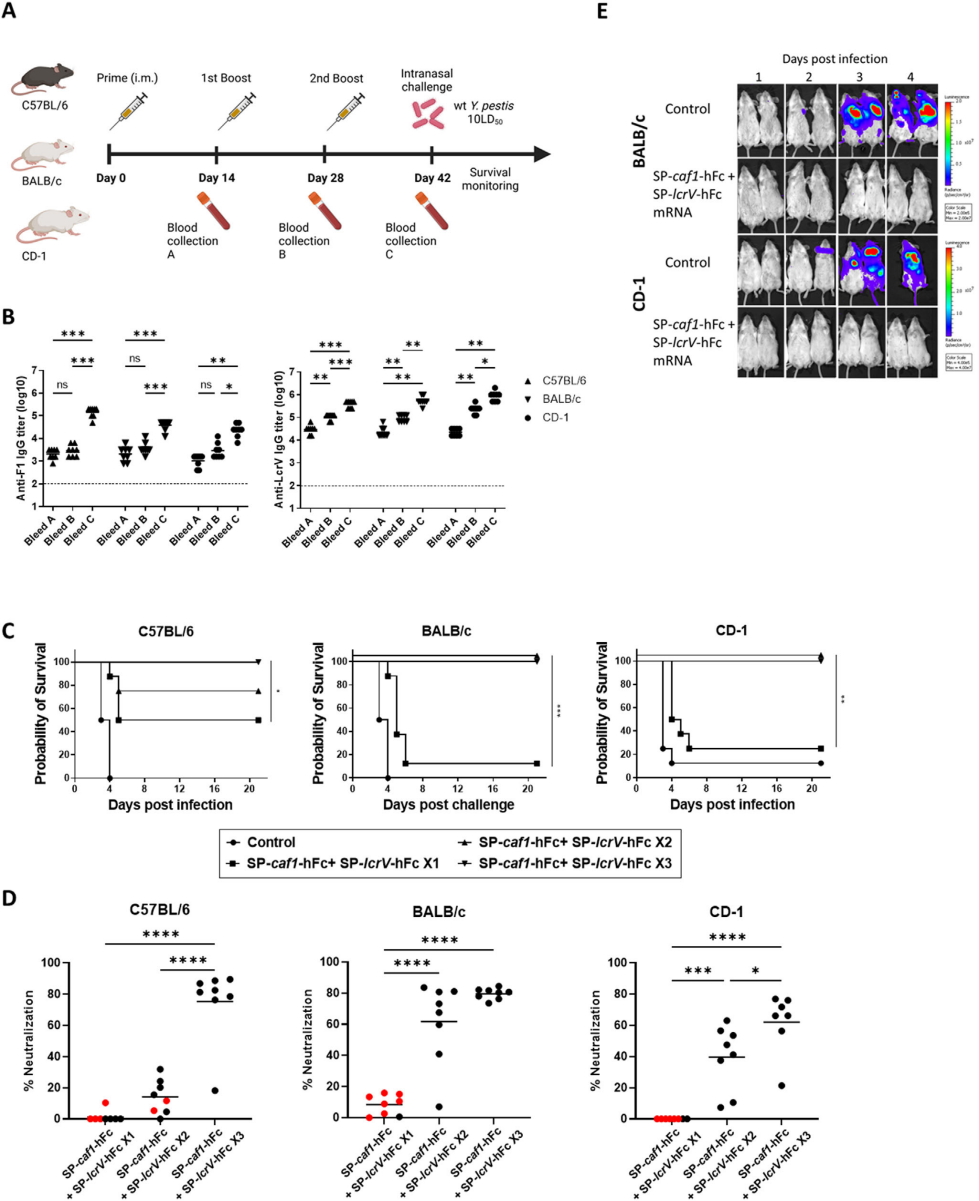
Immunogenicity and protective efficacy of bivalent F1 + LcrV mRNA‐LNPs against pneumonic *Y. pestis*. A) Schematic representation of vaccination regimen. C57BL/6, BALB/c and CD‐1 mice (*n* = 8) were vaccinated intramuscularly with a combination of SP‐*lcrV*‐hFc and SP‐*caf1*‐hFc (5µg each) once (day 28), twice (days 14, 28) or thrice (days 0, 14, 28). Blood samples were collected before each booster dose (A/B/C), and serum samples were assayed for anti‐LcrV and anti‐F1 IgG antibodies by ELISA (B). Two weeks after the last vaccination (day 42), animals were subjected to a lethal (10LD_50_) intranasal wt Kimberley53 challenge and monitored for survival (C). D) Sera from vaccinated animals were tested for cytotoxicity neutralization (% neutralization). Surviving animals are represented by black symbols and non‐surviving animals are represented by red symbols. E) IVIS imaging of *Y. pestis* dissemination following a lethal challenge in BALB/c and CD‐1 mice. BALB/c and CD‐1 mice were vaccinated intramuscularly with a combination of SP‐*lcrV*‐hFc and SP‐*caf1*‐hFc (5µg each) thrice (days 0, 14, 28). Two weeks after the last vaccination (day 42), animals were subjected to a lethal (10LD50) intranasal luciferase‐expressing Kimberley53 challenge and monitored for bacterial dissemination. Unvaccinated mice served as control. Statistical analysis was performed using a one‐way ANOVA with Tukey's multiple comparisons test (for immune responses and neutralizing assay) or log‐rank (Mantel‐Cox) test (for survival plot), (*p < 0.05, **p < 0.01, ***p < 0.001, and ****p < 0.0001). Dashed line indicates limit of detection for ELISA. The schematic for panel 5A was created with Biorender.

Having demonstrated the immunogenicity and protective efficacy of LcrV‐based mRNA vaccines, we next evaluated the effectiveness of a bivalent mRNA vaccine encoding both LcrV and our previously established F1 antigen mRNA construct. We have recently demonstrated the protective potential of an Fc‐conjugated F1 mRNA construct, SP‐*caf1*‐hFc, in the bubonic plague infection model.^[^ ^7^ ^]^ Given the high potency of the Fc‐conjugated LcrV mRNA construct observed in the current study against a pneumonic plague mouse model, we decided to use both F1 and LcrV in their Fc‐conjugated form for our bivalent vaccine (see construct design in Figure 1A). C57BL/6, BALB/c and CD‐1 mice were vaccinated once, twice or thrice at 2‐week intervals with a combination of SP‐*lcrV*‐hFc and SP‐*caf1*‐hFc mRNA (co‐administration of two LNP formulations) (see outline in **Figure** **5**A), and humoral response was monitored for anti‐LcrV and anti‐F1 IgG titers. Two weeks after the last administration, mice were challenged intranasally with a lethal dose (10LD_50_) of the fully‐virulent Kimberley53 *Y. pestis* strain, and monitored for survival. Figure 5B shows that the administration of three vaccination doses led to robust anti‐LcrV and anti‐F1 responses. Notably, the bivalent vaccine's high efficacy led to 100% survival after a three‐dose vaccination regimen, and this protective effect was sustained even with a two‐dose regimen, conferring 100% protection in BALB/c and CD‐1 mice and 75% protection in C57BL/6 mice (Figure 5C). Importantly, this high protective efficacy was generally in correlation with the neutralizing capacity of sera from vaccinated BALB/c and CD‐1 mice (Figure 5D). After the second vaccination, C57BL/6 mice displayed lower neutralizing capacity, suggesting that survival following the challenge may have been achieved primarily by the anti‐F1 antibody response. This was supported by the high levels of protection provided by prime‐boost vaccination of C57BL/6 mice with SP‐*caf1*‐hFc mRNA (Figure S1, Supporting Information). Shortening the vaccination regimen to a single dose compromised protective efficacy, resulting in survival rates of 50%, 12.5% and 25% in C57BL/6, BALB/c and CD‐1 mice, respectively, as also reflected by the low neutralization capacity of the sera (Figure 5C,D).

The ability to eliminate bacterial burden and survive lethal infection was further demonstrated using a lethal infection model with a luciferase‐expressing Kimberley53 *Y. pestis* strain allowing the visualization of bacterial dissemination and growth following the intranasal challenge. Whole body In Vivo Imaging System (IVIS), captured the bioluminescent signal from Luc‐expressing bacteria in naïve versus vaccinated mice. As can be seen in Figure 5E, luciferase signal was detectable as early as day 2 post‐infection in the lungs of control (unvaccinated) BALB/c and CD‐1 mice. By day 3, an intense and widespread signal indicated high bacterial burden and systemic dissemination, which continued to increase on day 4, after which animals succumbed to infection. In contrast, no luminescent signal was detected in vaccinated mice (receiving 3 doses of SP‐*lcrV*‐hFc and SP‐*caf1*‐hFc mRNA), indicating that the vaccination elicited robust immune responses that successfully cleared the bacteria.

**Figure 6:**
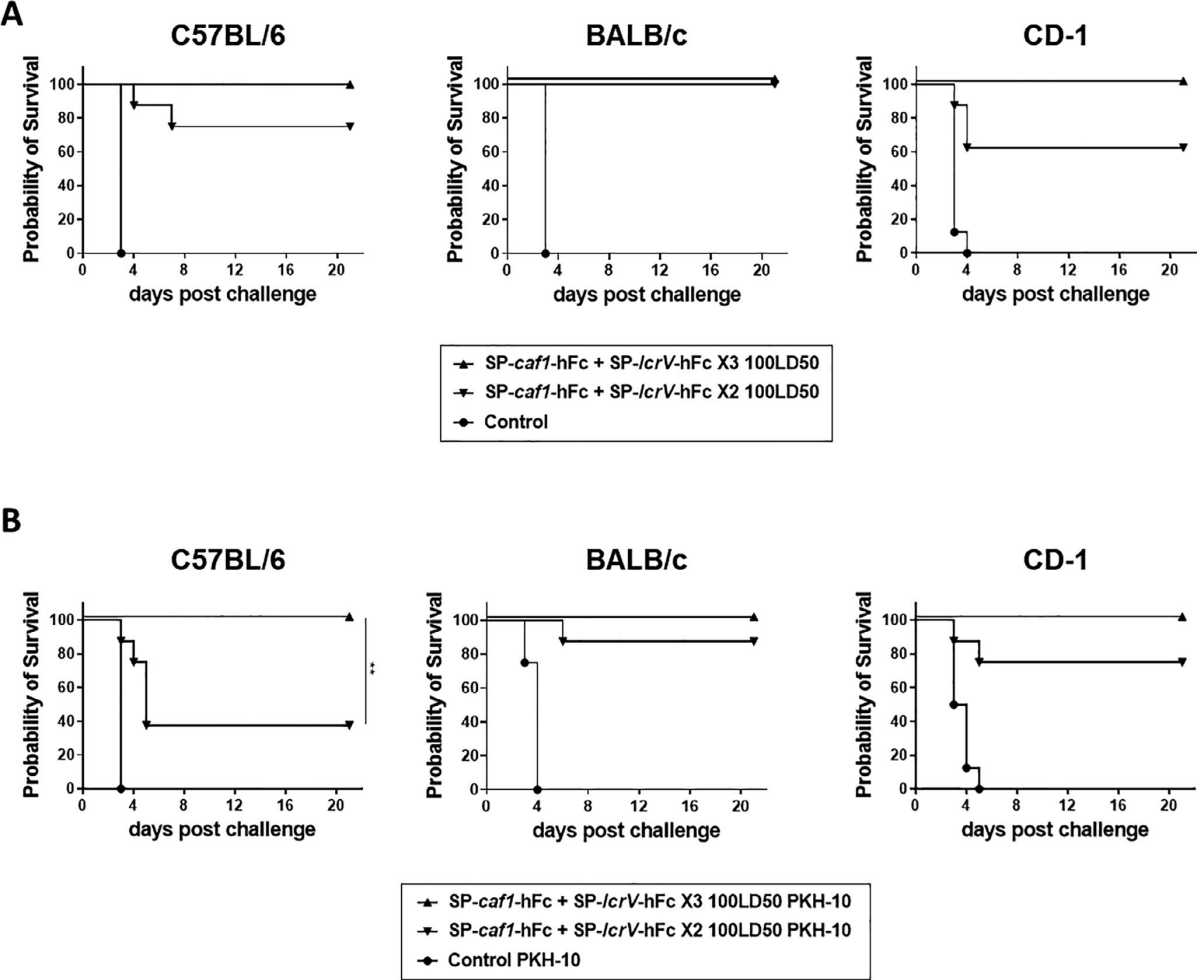
Protective efficacy of bivalent F1 + LcrV mRNA‐LNPs against high dose intranasal challenges with two different *Y. pestis* biovars. C57BL/6, BALB/c, and CD‐1 mice (*n* = 8) were vaccinated intramuscularly with 2 or 3 doses of a combination of SP‐*lcrV*‐hFc and SP‐*caf1*‐hFc (5µg each). Two weeks after the last vaccination (day 42), animals were subjected to high dose (100LD50) Kimberley53 (A) or PKH‐10 (B) challenges and monitored for survival. Statistical analysis was performed using log‐rank (Mantel‐Cox) test (for survival plot), (**p < 0.01).

To further examine the robustness of the bivalent vaccine, we next vaccinated mice with 2 or 3 doses of SP‐*lcrV*‐hFc and SP‐*caf1*‐hFc mRNA, and performed a high challenge dose (100LD_50_) with the wild‐type Kimberley53 *Y. pestis* strain. Mice of all strains vaccinated with three doses of the bivalent vaccine were completely protected against the high dose challenge. Shortening the immunization regimen to two doses still maintained high level of protective efficacy, with 75%, 100% and 62.5% survival in C57BL/6, BALB/c and CD‐1 mice, respectively (**Figure** **6**A). Next, we repeated the vaccination scheme described above, and conducted a high challenge dose (100LD_50_) using a different fully‐virulent *Y. pestis* strain, the PKH‐10 that belongs to the Medievalis biovar. Consistent with the results of the Kimberley53 challenge, full protection was recorded across all mouse strains that received 3 doses of the vaccine. In animals that were vaccinated twice, survival rates were significantly reduced (C57BL/6; 37.5%) or were only slightly reduced (BALB/c; 87.5%) and (CD‐1; 75%) (Figure 6B).

### 2.5 The LcrV Component of the Bivalent F1 + LcrV mRNA Vaccine Provides Protection Against an Unencapsulated *Y. pestis* Strain

**Figure 7:**
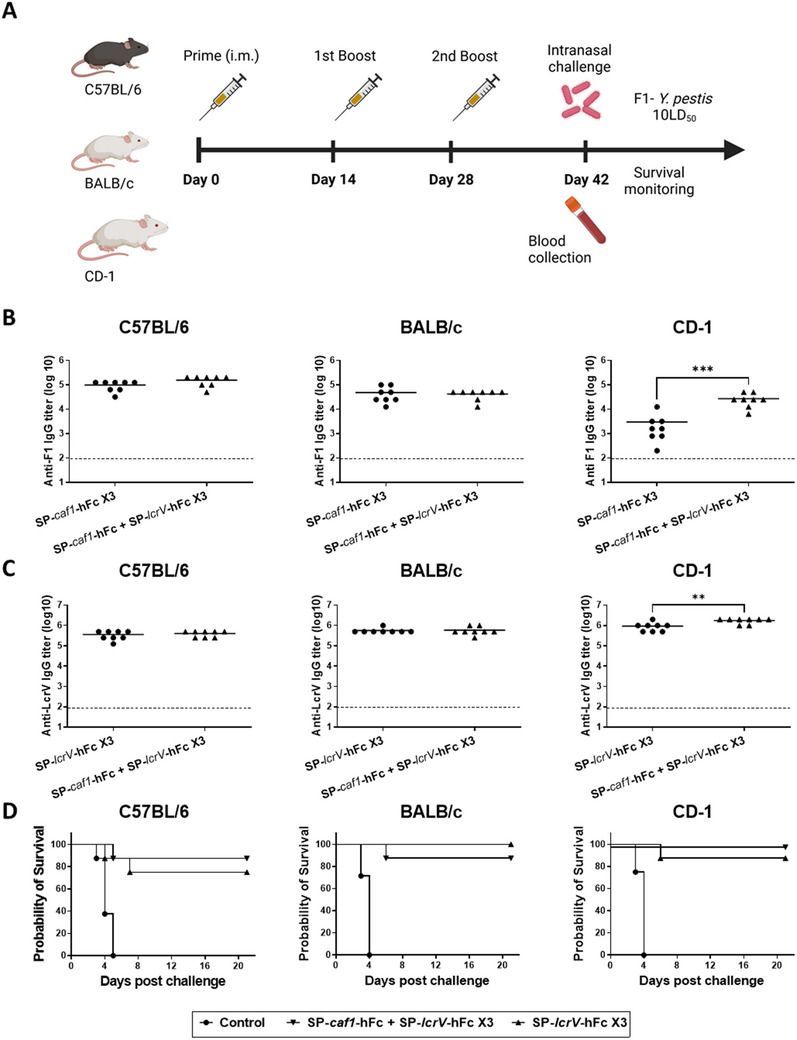
Immunogenicity and protective efficacy of bivalent F1 + LcrV mRNA‐LNPs against the unencapsulated F1^‐^ *Y. pestis* strain. A) Schematic representation of vaccination regimen. C57BL/6, BALB/c and CD‐1 mice (*n* = 8) were vaccinated intramuscularly with either SP‐*lcrV*‐hFc (5µg) or a combination of SP‐*lcrV*‐hFc and SP‐*caf1*‐hFc (5µg each) thrice (days 0, 14, 28). Blood samples were collected 14 days before the challenge, and serum samples were assayed for anti‐F1 (B) and anti‐LcrV (C) IgG antibodies by ELISA. Two weeks after the last vaccination (day 42), animals were subjected to a lethal (10LD50) intranasal challenge with the unencapsulated F1^−^ Kimberley53 strain and monitored for survival (D). Statistical analysis was performed using an unpaired student's *t*‐test (for immune responses) or log‐rank (Mantel‐Cox) test (for survival plot), (*p < 0.05, **p < 0.01, ***p < 0.001, and ****p < 0.0001). Dashed line indicates limit of detection for ELISA. The schematic for panel 6A was created with Biorender.

Finally, to examine the individual contribution of the LcrV and F1 antigens in the bivalent mRNA vaccine, we immunized C57BL/6, BALB/c and CD‐1 mice with three doses of mRNA coding for SP‐*caf1*‐hFc, SP‐*lcrV*‐hFc or a mixture of both of them (see experimental design in **Figure** **7**A). Anti‐LcrV and anti‐F1 IgG antibody titers were determined on day 41, one day prior to challenge (Figure 7B,C). Mice were then intranasally challenged with a lethal dose (10LD_50_) of the unencapsulated F1^‐^ Kimberley53 *Y. pestis* strain and monitored for 21 days. Vaccination with the bivalent mRNA vaccine did not hamper the antibody levels of either individually administered SP‐*caf1*‐hFc or SP‐*lcrV*‐hFc, and even increased the overall average anti‐F1 and anti‐LcrV antibody titer in CD‐1 mice, possibly due to a general adjuvant effect elicited by the combination of the two antigens. Since this strain does not express the F1 capsule antigen, all mice vaccinated with SP‐*caf1*‐hFc mRNA succumbed to infection (data not shown). Immunization with the bivalent mRNA vaccine provided high levels of protection across all mouse strains, with 87.5%, 87.5% and 100% survival rates in C57BL/6, BALB/c and CD‐1 mice, respectively (Figure 7D). As expected, animals vaccinated with SP‐*lcrV*‐hFc mRNA as a single antigen exhibited similar survival rates, with 75%, 100% and 87.5% survival in C57BL/6, BALB/c and CD‐1 mice, respectively. Taken together, these observations suggest that the protection afforded by the bivalent SP‐*lcrV*‐hFc + SP‐*caf1*‐hFc mRNA vaccine against the F1^‐^ Kimberley53 strain is primarily attributable to the LcrV component of the bivalent vaccine.

## 3 Discussion

Despite extensive global efforts over several decades to develop an effective and safe vaccine against *Yersinia pestis*, none has been approved to date in Western countries for protection against this notorious bacterial pathogen.^[^ ^16^ ^]^ This gap in protection is particularly concerning given that *Y. pestis* is classified as a Tier 1 biothreat agent due to its ability to cause plague – a severe and often fatal disease.^[^ ^17^ ^]^ Until recently, preclinical immunization studies have focused primarily on live‐attenuated or adjuvant‐formulated protein subunit vaccines, with F1 and LcrV emerging as the principal antigens under investigation.^[^ ^6^, ^16^, ^18^, ^19^, ^20^, ^21^, ^22^, ^23^, ^24^, ^25^, ^26^, ^27^, ^28^, ^29^ ^]^ Both F1 and LcrV antigens have undergone clinical trials to evaluate their safety and immunogenicity, underscoring their potential for plague prophylaxis.^[^ ^30^, ^31^, ^32^ ^]^

Recent advances in mRNA vaccine technology, highlighted by the success of SARS‐CoV‐2 mRNA vaccines, have propelled this novel platform to the forefront of vaccine research. mRNA‐based vaccines have already been explored for use against a broader range of pathogens,^[^ ^33^, ^34^, ^35^, ^36^ ^]^ emphasizing the platform's adaptability for rapid vaccine development in diverse infectious and biothreat contexts.

Our earlier work demonstrated the feasibility of applying this platform to bacterial pathogens, as evidenced by the design and evaluation of an effective F1‐based mRNA vaccine in a mouse model of bubonic plague caused by *Y. pestis.^[^* ^7^ *^]^* In the current study, we aimed to assess the protective potential of a bivalent mRNA vaccine incorporating both F1 and LcrV components to address the pneumonic form of plague, a rapidly progressing infectious bacterial disease with high mortality and significant public health implications, and to overcome challenges posed by non‐encapsulated *Y. pestis* strains lacking F1.^[^ ^17^ ^]^ For this purpose, we initially designed two mRNA constructs: one encoding the native LcrV antigen (SP‐*lcrV*), and another encoding a human Fc‐conjugated LcrV (SP‐*lcrV*‐hFc). The rationale for hFc conjugation stemmed from our previous findings, where conjugating the F1 antigen to hFc significantly enhanced immunogenicity and protective efficacy.^[^ ^7^ ^]^ In addition, Fc conjugation is known to improve the pharmacokinetic and immunogenic properties of proteins, including increased half‐life, enhanced solubility, improved adjuvanticity, and more efficient delivery.^[^ ^10^, ^11^, ^12^, ^37^ ^]^ The success of Fc fusion technology is further demonstrated by the regulatory approval of numerous Fc‐fusion products for clinical use by the FDA and EMA, underscoring its potential in vaccine development and other therapeutic applications.^[^ ^38^ ^]^

Our results demonstrated that the SP‐*lcrV*‐hFc mRNA construct provided full protection against pneumonic plague across all three tested mouse strains, including inbred C57BL/6, BALB/c, and outbred CD‐1 mice. In contrast, the SP‐*lcrV* mRNA construct lacking the Fc domain was protective in BALB/c and CD‐1 mice but largely ineffective in C57BL/6 mice, with nearly all animals succumbing to infection. These findings suggest that Fc‐conjugation enhances immune responses to LcrV, likely by improving antigen stability and activating Fc‐receptor‐mediated pathways. This underscores how vaccine design can leverage Fc interactions to enhance both innate and adaptive immunity, a principle that could be applied toward other bacterial vaccine targets.

Notably, although the protective efficacy varied between LcrV constructs, we observed similar and robust anti‐LcrV binding antibody response across all mouse strains, regardless of the type of vaccination, aligning with prior reports that anti‐LcrV binding antibody titers alone are not reliable correlates of protection.^[^ ^14^, ^39^, ^40^, ^41^, ^42^ ^]^ The differential efficacy of the LcrV mRNA constructs across mouse strains points to the complexity of vaccine‐induced immune responses and their dependence on genetic background.

To further explore immune correlates of protection, we employed our previously developed macrophage cytotoxicity neutralization assay to quantify the capacity of antibodies from vaccinated animals to neutralize the cytotoxic effects of *Y. pestis*.^[^ ^14^ ^]^ In BALB/c and CD‐1 mice, high levels of neutralization (>70%) were observed in most vaccinated animals (31 out of 47), regardless of the construct used, correlating with robust protection. However, in C57BL/6 mice, vaccination with SP‐*lcrV* mRNA elicited minimal neutralizing antibody levels (0–20%) in most animals, resulting in poor survival rates, with only 1 out of 8 mice surviving infection. Interestingly, recombinant LcrV showed improved efficacy in this strain, with 5 out of 8 mice achieving >60% neutralization and surviving the challenge. Notably, SP‐*lcrV*‐hFc vaccination conferred complete protection in C57BL/6 mice, even though 37.5% of animals displayed low neutralizing potential (0–20%). This underscores the importance of exploring additional immune mechanisms, such as Fc‐receptor engagement, effector cell recruitment, or T‐cell support, which could compensate for low neutralizing antibody titers.

Evaluation of antigen‐specific IgG subclasses revealed a consistent trend across all three mouse strains. As expected, vaccination with the alum‐adjuvanted recombinant LcrV elicited a higher IgG1 response, indicative of a T_H_2‐biased humoral immune response.^[^ ^15^, ^43^ ^]^ In contrast, both SP‐*lcrV* and SP‐*lcrV*‐hFc mRNA vaccines produced an IgG2a/IgG1 or IgG2c/IgG1 ratio of ≈1, suggesting a more balanced T_H_1/T_H_2 response that fosters stronger cellular immunity. Such a dual humoral–cellular profile is especially valuable for targeting intracellular stages of *Y. pestis* infection and may inform broader strategies in bacterial vaccine design. ELISpot analysis further supported this enhanced cell‐mediated immunity by revealing robust IFN‐γ release in splenocytes from SP‐*lcrV* and SP‐*lcrV‐*hFc mRNA–vaccinated mice, compared to negligible responses in those vaccinated with the recombinant protein. Notably, BALB/c and CD‐1 mice exhibited a significantly higher cellular response than C57BL/6 mice, which may explain the superior efficacy of SP‐*lcrV* mRNA vaccination observed in these two strains.

Given its high protective efficacy against pneumonic plague, the SP‐*lcrV*‐hFc construct was selected for subsequent experiments to evaluate a bivalent mRNA vaccine against *Y. pestis*. This vaccine combined SP‐*lcrV*‐hFc with a human Fc‐conjugated F1‐based mRNA construct (SP‐*caf1*‐hFc), previously shown to be highly effective against bubonic plague.^[^ ^7^ ^]^ The resulting vaccine conferred robust protection against severe bacterial pneumonia in multiple mouse strains challenged with two different *Y. pestis* biovars (Orientalis and Medievalis). Notably, it achieved high efficacy even with a two‐dose regimen, underscoring its capacity to rapidly induce a potent immune response in line with World Health Organization (WHO) target product profile (TPP) requirements for plague vaccines.^[^ ^44^ ^]^ Given that *Y. pestis* is a Tier 1 biothreat agent, the adaptability of the mRNA platform extends beyond conventional infectious disease control to biodefense applications.

Notably, both the bivalent formulation and the single‐component SP‐*lcrV*‐hFc vaccine also protected against an F1‐negative *Y. pestis* strain. However, considering the possible polymorphism within the LcrV protein,^[^ ^45^, ^46^ ^]^ incorporating additional antigens into the vaccine will ensure broader and more reliable protection. An elegant study by Chopra et al. demonstrated the incorporation of a third antigen, the T3SS needle protein YscF, into a trivalent adenoviral vector (rAd5‐YFV) encoding F1, LcrV, and YscF, which demonstrated superior protection against bubonic and pneumonic plague compared to a monovalent LcrV‐encoding vector.^[^ ^47^ ^]^ The flexible mRNA platform described in our study could similarly integrate additional or alternative antigens to generate multivalent vaccines capable of countering antigenic variations in *Y. pestis* and other pathogens.

Interestingly, LcrV shares functional similarity with PcrV, a key component of the type III secretion system (T3SS) in *Pseudomonas aeruginosa*, and recent work has shown that mRNA vaccines encoding PcrV and OprF provide robust protection against multiple *Pseudomonas* strains.^[^ ^48^ ^]^ Likewise, a multivalent mRNA‐LNP vaccine targeting *Clostridioides difficile* toxins and virulence factors elicited durable systemic and mucosal responses, effectively protecting mice from lethal infection.^[^ ^33^ ^]^ Additionally, a self‐amplifying RNA (saRNA) vaccine encoding *Y. pestis* F1 and LcrV has been reported demonstrating immunogenicity in both BALB/c and outbred OF‐1 mice and conferring 70–80% protection against bubonic plague.^[^ ^49^ ^]^ These findings underscore the broad potential of mRNA‐based platforms in countering diverse bacterial threats through the inclusion of multiple antigens. By eliciting robust humoral and cellular immunity and reducing reliance on antibiotics, this approach also aligns with efforts to combat antimicrobial resistance.

## 4 Study Limitations

We recognize several limitations in the current study. While robust immunogenicity and protective efficacy were demonstrated in three different mouse models, these results may not directly translate to humans, as differences in genetic background, immune responses, and physiology could affect vaccine efficacy. Future studies should explore additional animal models, including non‐human primates, to improve translational relevance. In addition, it appears that the genetic background of the animals is the most influential factors in determining protective efficacy especially for the SP‐*lcrV* mRNA construct. The exact causes remain unclear but can result from differences in protein expression levels, immune presentation, and RNA/protein stability. Further studies are needed to clarify these issues and better understand the mechanisms influencing vaccine efficacy. Lastly, our primary focus was to demonstrate that vaccination provides a rapid prophylactic solution against pneumonic plague, which is crucial for containing localized outbreaks, as plague can be transmitted from person to person. Establishing rapid protection was a key first step in vaccine development. Future studies will evaluate the longevity of the immune response to determine the duration of protection and its potential for long‐term immunity. Addressing these limitations will be critical for translating this vaccine into a viable human plague prophylactic.″

This study establishes the mRNA‐LNP platform as a promising tool for addressing bacterial pathogens, including those resistant to antibiotics. By broadening its applicability to diverse threats, this technology represents an innovative approach to tackling some of the most pressing challenges in global health. Rapid manufacturing and modular antigen selection can facilitate immediate responses to novel or engineered threats, a pivotal advantage in pandemic preparedness. Future studies should optimize dosing regimens, assess long‐term immunity, and evaluate vaccine efficacy against broader pathogen spectra and in additional animal models to fully realize the potential of mRNA vaccines in preventing bacterial diseases.

## 5 Experimental Section

### Materials and Chemicals

Cholesterol, distearoyl‐*sn*‐glycero‐3‐phosphocholine (DSPC), and dimyristoyl‐rac‐glycero‐3‐methoxypolyethylene glycol (DMG‐PEG) were from Avanti Polar Lipids (Alabaster, AL, USA). The proprietary ionizable lipid (lipid 14) was synthesized in‐house as previously published.^[^ ^13^ ^]^ mRNA sequences were purchased from TriLink (San Diego, CA, USA) or synthesized by an in vitro transcription (IVT) reaction using the MEGAscript T7 transcription kit and cleaned by the MEGAclear transcription clean‐up kit both from Thermo Fisher Scientific (Waltham, MA, USA). All mRNA sequences were synthesized with complete N1‐methyl‐pseudouridine nucleotide substitution (Hongene Biotech).

### Design of mRNA constructs

The mRNA construct encoding for the hFc‐conjugated monomeric F1 (SP‐*caf1*‐hFc) were previously described in ref. [7] Two mRNA constructs coding for the low‐calcium response virulence (LcrV) antigen of *Yersinia pestis* were designed as follows: SP‐*lcrV*; The lcrv gene (GenBank, account number KF682423.1) section coding for Ile2 to Lys326 was preceded by the signal peptide sequence originating from the immunoglobulin (Ig) light chain variable region (GenBank, account number U43767.1), SP‐*lcrV*‐hFc is composed of the SP‐*lcrV* sequence, followed by the sequence coding for the constant region of the human IgG1 (adopted from GenBank account number AEV43323.1). All mRNA constructs were codon optimized for expression in mice, included an initiator methionine and a Kozak consensus sequence.

### LNP Preparation and Characterization

Ionizable lipid, cholesterol, DSPC, and DMG‐PEG were mixed at a molar ratio of 40:47.5:10.5:2 with absolute ethanol in a tube. mRNA payloads were suspended in citrate buffer (50 mm, pH 4.5). To create LNPs, a dual‐syringe pump was used to transport the two solutions through the NanoAssembler micromixer from Precision NanoSystem (Vancouver, British Columbia, Canada) at a total flow rate of 12 mL min^−1^. The particles were then transferred into dialysis overnight against PBS. Particles in PBS were analyzed for size and uniformity by dynamic light scattering (DLS).

### Cell Transfection and Western Blotting

One day before the transfection, HeLa cells (10^5^ cells/well) were seeded in 24‐well plates (Costar). At the day of the transfection, in vitro transcribed and purified mRNA (0.5 µg) coding for SP‐*lcrV* or SP‐*lcrV*‐hFc was mixed with Lipofectamine MessengerMax transfection reagent (Invitrogen) and added to each well. Control cells were not transfected. 48 to 72 h afterwards the cells were harvested and the supernatant was separated from the cells by centrifugation. Cell pellets were washed once with phosphate‐buffered saline (PBS) and lysed with radioimmunoprecipitation assay (RIPA) lysis buffer (Merck). Equal amounts of protein were resuspended in 1x Laemmli sample buffer (Bio‐Rad) supplemented with dithiothreitol (DTT), boiled and resolved by 10% SDS–PAGE. Resolved proteins were blotted to iBlot mini nitrocellulose membranes (Thermo Fisher Scientific). LcrV antigen expression was detected by in‐house rabbit anti‐LcrV antibodies, followed by incubation with fluorophore‐conjugated goat anti‐rabbit antibodies (IRDye® 800CW; LI‐COR). Immune complexes were detected by using the ODYSSEY CLx imager and the coupled Image Studio™ Software (LI‐COR).

### Ethics Statement

This study was carried out in strict accordance with the recommendations for the Care and Use of Laboratory Animals of the National Institutes of Health. All animal experiments were performed in accordance with Israeli law and were approved by the Ethics Committee for animal experiments at the IIBR (permit numbers M‐15‐23, M‐33‐23, M‐40‐23, M43‐23, and M‐26‐24). During the experiments, the mice were monitored daily. Humane end points were used in our survival studies. Mice exhibiting loss of the righting reflex were euthanized by cervical dislocation.

### Bacterial Strains

The *Y. pestis* strains used in this study are listed in **Table** **1**. Maintenance of the virulence‐associated plasmids pMT1, pCD1, pPCP1 and the *pgm* locus was verified by PCR analysis in all of the virulent *Y. pestis* strains. *Y. pestis* strains were routinely grown on brain heart infusion agar (BHIA, Difco) for 48 h at 28 °C. The ampicillin‐resistant strain EV76ΔJ+P and Kimberley53‐Lux were grown on BHIA supplemented with ampicillin (100 mg mL^−1^, Sigma, Israel). Construction of the F1^‐^ Kimberley53 mutant was performed by replacing part of the *caf1* gene coding sequence with a linear fragment containing the Kan^R^ GeneBlock™ resistance cassette (pUC4K plasmid, Pharmacia) by homologous recombination, using previously established methodologies.^[^ ^50^ ^]^ The knockout phenotype of this mutant strain was verified by PCR, whole genome sequencing and by Western blot.

**Table 1:** *Y. pestis* strains used in this study.

Strain	Relevant characteristics	Refs.
Kimberley53 (Kim53)	Virulent strain, biovar Orientalis	[21, 51, 52]
Kimberley53 F1^‐^	Virulent *caf1*‐deleted Kim53	This study
Kimberley53‐Lux	Virulent bioluminescent Kim53 derivative carrying the pGEN‐luxCDABE plasmid	[52, 53, 54]
PKH‐10	Virulent strain, biovar Medievalis	[55]
EV76‐ ΔyopJ+pyopP	Avirulent, yopJ‐deleted EV76 over‐expressing YopP of *Y. enterocolitica* WA 0:8	[14]

### Animals

Female and male C57BL/6JOlaHsd mice (6 to 8 weeks old) were obtained from Harlan (Israel). BALB/c and CD‐1 female mice were purchased from Charles River (UK). All mice were randomly assigned into cages in groups of 10 animals. The mice were allowed free access to water and rodent diet (Harlan, Israel).

### Animal Vaccination Experiments

The vaccination regimes examined in this study included one, two and three doses administered in a 14‐day interval. In each dose the animals were vaccinated intramuscularly (50 µl to each hind leg muscle, for a total of 100 µl) with SP‐*lcrV*, SP‐*lcrV*‐hFc, SP‐*caf1*‐hFc mRNA‐LNPs (5 µg), or PBS (negative control). Aluminum‐hydroxide (AlOH) gel (Brennentag Biosector, Denmark)‐adsorbed V antigen protein (20 µg per mouse per dose) and aluminum‐hydroxide (AlOH; 0.36% final concentration per mouse per dose) were similarly administered as positive and negative controls, respectively. Blood samples were taken from the tail vein before every boost and just prior to the challenge. Throughout the vaccination experiments, mice were monitored for gross visible physical and behavioral signs that could indicate possible vaccine‐related side effects, and these were not detected.

### Anti‐V and Anti‐F1 Enzyme‐Linked Immunosorbent Assay

Flat‐bottom Maxisorp 96‐well microtiter plates (Thermo Fisher Scientific) were coated with purified LcrV antigen (350 ng) or polymeric F1 [500 ng, provided by the Biotechnology Department at the Israel Institute for Biological Research (IIBR)] in 1xPBS. Sera of vaccinated animals was diluted in PBS (x1), bovine serum albumin (2%), and Tween 20 (0.05%), and serially diluted in twofold dilutions in a final volume of 50 µl. Alkaline phosphatase–conjugated goat anti‐mouse IgG (1/4000 dilution; Sigma‐Aldrich) was used as the second layer for anti‐LcrV IgG or anti‐F1 IgG titer determination. All incubation steps were performed for 1 h at 37 °C. The plates were extensively washed with PBS (1×) and Tween 20 (0.05%) before the incubation steps. Titers were defined as the reciprocal values of the end point serum dilutions that displayed optical density at 405 nm (OD405) values twofold higher than the normal serum controls obtained from naïve animals. Standardization of the titer value was achieved by including serial twofold dilutions of a purified monoclonal anti‐LcrV antibody^[^ ^56^ ^]^ or of mouse anti‐F1 polyclonal serum in each of the test plates. The IgG1, IgG2a, and IgG2c isotype titers of the anti‐LcrV antibodies were determined using isotype‐specific secondary antibodies (SouthernBiotech).

### Macrophage Cytotoxicity Neutralization Assay

The EV76 derivative *Y. pestis* ΔyopJ + yopP that was previously described^[^ ^14^ ^]^ was seeded on brain heart infusion agar plates (BHIA, Difco). 48 h later several *Y. pestis* colonies were grown at 28 °C in heart infusion broth (HIB) for 18 h at 150 rpm. Bacterial cultures were then diluted in HIB medium to an initial OD_660_ of 0.05, and allowed to grow for 1 h at 26 °C (100 rpm) and for 2 additional hours at 37 °C (100 rpm). Under these conditions Yops are expressed and primed for translocation into phagocytic cells, yet bacteria do not express the F1 capsule antigen. Bacteria were collected by centrifugation, washed, and re‐suspended in DMEM supplemented with 10% fetal calf serum (FCS) to a concentration of 2 × 10^6^ CFU mL^−1^ and then used to infect J774A.1 cells (2 × 10^4^ cells/well in 96‐well tissue culture plates). In neutralization assays, heat‐inactivated sera were added to aliquots of bacterial suspensions at a dilution of 1:40, incubated for 15 min at 37 °C and added to the cultured macrophages at a multiplicity of infection (MOI) of 10. Immediately afterwards the plates were centrifuged for 5 min at 130 g, to promote uniform infection, and incubated at 37 °C, 5% CO_2_. Thirty minutes later gentamicin was added (50 µg mL^−1^) and the cells were further incubated for additional 6 h at 37 °C, 5% CO_2_. Lactate dehydrogenase (LDH) levels in the cell supernatants of infected macrophages were determined using the Cytotox 96® non‐radioactive cytotoxicity assay (Promega) according to the manufacturer's instructions; absorbance (A490) was determined using a microplate reader (SpectraMax ABS Plus; Molecular Devices). The percentage of cell death was determined according to the formula: 100 × (experimental release − spontaneous release)/ (maximum release − spontaneous release). The spontaneous release reflects the amount of LDH released from the cytoplasm of uninfected macrophages. In standard cytotoxicity assays, the maximal release is the amount of LDH released upon treatment with detergent (as recommended by the kit manufacturer), whereas in neutralization assays the maximal release was defined as the amount of LDH released from cells infected with bacteria incubated with control mouse sera. All experiments were performed at least three times.

### Murine Interferon‐γ Enzyme‐Linked Immunosorbent Spot Assay

Mouse spleen was dissociated in a gentleMACS C tube (Miltenyi Biotec), filtered, treated with red blood cell lysing buffer (SigmaAldrich), and washed. Pellets were resuspended in 1 mL of CTL‐Test medium [Cellular Technology Limited (CTL) supplemented with 1% fresh glutamine and 1 mm P/S (Biological Industries, Israel), and single‐cell suspensions were seeded onto 96‐well, high‐protein‐binding, polyvinylidene difluoride filter plates at 4 × 10^5^ cells per well. Mice were tested individually in duplicates by stimulation with a 15‐mer peptide library covering the LcrV coding sequence (10 µg mL^−1^) (GenScript), concanavalin A (2 µg mL^−1^; Sigma‐Aldrich) as positive control, or CTL medium as negative control (no antigen). Cells were incubated at 37 °C, 5% CO_2_ with antigens for 24 h, and the frequency of interferon‐γ (IFN‐γ)–secreting cells was determined using a murine IFN‐γ single color enzymatic enzyme‐linked immunosorbent spot (ELISpot) kit (CTL, no. MIFNG 1M/5) with strict adherence to the manufacturer's instructions. Spot‐forming units were counted using an automated ELISpot counter (CTL).

### Mouse Infection

Intranasal (i.n.) infections were performed as described previously in.^[^ ^25^ ^]^ Briefly, a loop full of typical *Y. pestis* Kimberley53, Kimberley53 F1^‐^ and PKH‐10 bacterial colonies was harvested and diluted in heart infusion broth (HIB, Difco) supplemented with xylose (0.2%) and CaCl_2_ (2.5 mm, Sigma‐Aldrich, St. Louis, MO, USA) to an OD_660_ of 0.01 and grown for 22 h at 28 °C and 100 rpm. At the end of the incubation period (OD_660_ ≈ 4), the culture was washed and diluted in saline (0.9% NaCl) to the desired infectious dose that was verified by counting colony forming units after plating and incubating on BHIA plates (48 h at 28 °C). Mice were anesthetized with a mixture of ketamine HCl (0.5%) and xylazine (0.1%) and then infected intranasally with 35 µL per mouse of bacterial suspension. The intranasal LD_50_ of the three fully‐virulent *Y. pestis* strains toward the C57BL/6 and BALB/c inbred mice is 1100 cfu and 2100 cfu toward CD‐1 mice. The LD_50_ values were calculated according to the method described by Reed and Muench.^[^ ^57^ ^]^ Mouse morbidity and mortality were monitored on a daily basis for 21 days. At the end of this period, clearance of the pathogen was verified by plating spleen homogenates from surviving animals onto selective BIN plates^[^ ^58^ ^]^ for 48 h at 28 °C.

### Statistical Analyses

All values are presented as means + SEM. Statistical analysis was performed using either a one‐way analysis of variance (ANOVA) with Tukey's multiple comparisons test or unpaired student's t‐test (for ELISA and neutralization data), a two‐way ANOVA followed by Bonferroni post hoc test (for ELISpot data) and a log‐rank (Mantel‐Cox) test (for survival data) (*p < 0.05, **p < 0.01, ***p < 0.001, and ****p < 0.0001). Sample sizes (n) are mentioned on each figure captions. All statistical analyses were performed using GraphPad Prism 9.4.1 statistical software.

## Conflict of Interest

D.P. receives licensing fees (to patents on which he was an inventor) from, invested in, consults (or on scientific advisory boards or boards of directors) or Founder and hold shares or conducts sponsored research at TAU for the following entities: ART Biosciences, BioNTech SE, Earli Inc., LAND Medicine Inc., Kernal Biologics, Merck, Newphase Ltd., NeoVac Ltd., RiboX Therapeutics, Roche, SirTLabs Corporation, Teva Pharmaceuticals Inc. All other authors declare no cnflict of interest.

## Supporting information



Supporting Information

## Data Availability

The data that support the findings of this study are available from the corresponding author upon reasonable request.
