# Supplementary material for: Novel Bivalent mRNA‐LNP Vaccine for Highly Effective Protection against Pneumonic Plague
Source: Adv Sci (Weinh). 2025 Apr 25;12(26):2501286. doi: 10.1002/advs.202501286 (PMC12245056; doi:10.1002/advs.202501286)
Supplement: Supplementary file 1 — Supporting Information [file ADVS-12-2501286-s001.docx]

**Supporting information**

**Novel Bivalent mRNA-LNP Vaccine for Highly Effective Protection Against Pneumonic Plague**

Uri Elia^1,7^, Yinon Levy^1,7^, Hila Cohen^1^, Ayelet Zauberman^1^, David Gur^1^, Inbal Hazan-Halevy^3,4,5,6^, Moshe Aftalion^1^, Shani Benarroch^3,4,5,6^, Erez Bar-Haim^1^, Orit Redy-Keisar^2^, Ofer Cohen^1,8^, Dan Peer^3,4,5,6,8^*, Emanuelle Mamroud ^1,8^

**Figure S1 - Protective efficacy of SP-caf1-hFc mRNA-LNPs against pneumonic Y. pestis.** C57BL/6 mice (n=16) were vaccinated intramuscularly with two doses of SP-caf1-hFc (5μg). Two weeks after the last vaccination, animals were subjected to a lethal (10LD_50_) intranasal Kimberley53 challenge and monitored for survival. Statistical analysis was performed using log-rank (Mantel-Cox) test (for survival plot), (****P < 0.0001).
